# Low dose versus standard dose rituximab for the treatment of antiphospholipid syndrome: A pilot study from a tertiary medical center

**DOI:** 10.3389/fimmu.2022.971366

**Published:** 2022-11-03

**Authors:** Yuzhou Gan, Xue Zhong, Yawei Zhao, Gongming Li, Hua Ye, Chun Li

**Affiliations:** ^1^ Department of Rheumatology & Immunology, Beijing Key Laboratory for Rheumatism and Immune Diagnosis (BZ0135), Peking University People’s Hospital, Beijing, China; ^2^ Center of Clinical Immunology, Peking University, Beijing, China; ^3^ Department of Pharmacy, Peking University People’s Hospital, Beijing, China; ^4^ Department of Rheumatology and Immunology, Luohe Central Hospital, Luohe, Henan, China; ^5^ Department of Rheumatology, Linyi Traditional Chinese Medicine Hospital, Linyi, Shandong, China

**Keywords:** rituximab, antiphospholipid syndrome, standard dose, low dose, real-world study

## Abstract

**Background:**

To investigate the therapeutic effects and safety of low-dose and standard-dose rituximab (RTX) in the treatment of antiphospholipid syndrome (APS).

**Methods:**

In this real-world study, we included 22 consecutive patients with APS who received RTX. Standard dose (SD) was defined as an overall dosage of RTX ≥ 1000mg in the induction period, and low dose (LD) was defined as an overall dosage of RTX <1000mg.

**Results:**

Of included patients, 1 patients died, 2 patients withdrew and 19 patients completed 6-month follow-up. Nine patients received SD-RTX and 13 patients received LD-RTX, and elder patients [LD-RTX vs. SD-RTX: (49.1 ± 15.5) vs. (35.8 ± 12.3) years, *p* = 0.044] and patients with later-onset [LD-RTX vs. SD-RTX: (46.8 ± 16.3) vs. (31.3 ± 13.6) years, *p* = 0.029] were more frequently included in LD-RTX than SD-RTX. Following 6 month RTX treatment, 8 patients (42.1%) achieved complete remission, 8 patients (42.1%) achieved partial remission and 3 patients (15.8%) showed no remission. The titers of anticardiolipin antibodies [baseline vs. 6 months: 30.8 (10.7, 90) vs. 19.5 (2.45, 69.10) U/L, *p* = 0.023] and the levels of erythrocyte sedimentation rate [baseline vs. 6 months: 29 (6, 63) vs. '6 (3, 14) mm/h, *p* = 0.021] exhibited a significantly decrease in all APS patients. Remission rate and titers of anti-β2-glycoprotein I and lupus anticoagulant did not differ significantly between two groups.

**Conclusion:**

RTX might be a safe and effective option for patients with APS, and low dose confers equal efficacy as standard dose. Further cohort studies are needed to confirm our findings.

## Introduction

Antiphospholipid syndrome (APS) is systemic autoimmune disease characterized by combination of vascular thrombosis, obstetrical complications and persistent presence of circulating antiphospholipid antibodies (aPLs) such as anti-β2-glycoprotein I (aβ2GPI), anticardiolipin antibodies (aCL) and lupus anticoagulant (LAC) (1). Up to now, the first-line treatment of APS consists of aspirin, low-molecular-weight heparin or warfarin. However, antithrombotic strategies are usually not effective for nonthrombotic manifestations, nephropathy and microthrombosis. On the basis of newly understood immunological mechanisms, immunomodulatory approaches targeting mTOR, B cells, and complement have been proposed as an add-on treatment in APS patients (2, 3).

As B-cells play an important role in APS pathophysiology, elimination of B cells could be a promising treatment option. Rituximab (RTX) is an anti-CD20 monoclonal antibody, which ultimately results in B-cell depletion and dysfunction (4). Although there are no controlled studies to compare the efficacy between RTX and placebo, the experience of RTX in the treatment APS has been proven by some observational studies in the last few years (5–8). For example, Doruk E. et al. and Sciascia S. et al. found RTX may be effective in controlling some non-criteria manifestations (thrombocytopenia, skin ulcer and cognitive dysfunction) regardless of substantial change in aPL profiles (8, 9). A multicentre Israeli study revealed complete response was associated with a decrease in aPL titers within 4–6 months after RTX treatment (7). To sum up, RTX may be an efficient treatment in APS, especially in controlling non-criteria manifestations.

Nevertheless, the safety of the long term and high dose RTX is the main concern. As we all know, the side effects of RTX included infections, allergy, infusion reaction, and so on (10). Recently, several studies have suggested that a low-dose regimen were closely similar to the successful results obtained with conventional regimens in other autoimmune diseases (10–14). In addition, a significantly lower incidence of infections and substantial costs saving were seen with low dose RTX (LD-RTX) compared with standard dose (12, 14). These outcomes indicated that high dose RTX might not be necessary for all the patients with autoimmune diseases and low dose RTX could be a promising new option with a satisfactory response rate.

However, the efficacy of low-dose RTX remains elusive in APS. The aim of our pilot study is to investigate the efficacy and safety of low-dose and standard-dose RTX for APS patients.

## Methods

### Patients

This is a real-world study in Department of Rheumatology and Immunology, Peking University People’s Hospital, based on our dynamic retrospective cohort of APS between July 2009 and January 2021. The diagnosis of APS was confirmed by two rheumatologists (YZG and CL) according to the 2006 revised Sapporo criteria (15) or catastrophic antiphospholipid syndrome (CAPS) according to the current diagnostic criteria (16). Another inclusion criterion was disease onset age ≥ 18 years. This study was approved by the ethics committee of Peking University People’s Hospital (2019PHB253-01) and complied with the Declaration of Helsinki guidelines. Written informed consent was obtained from each patient.

### Clinical and laboratory data collection

Baseline data were obtained from the electronic medical records before the initial RTX treatment, including demographics, duration of symptoms, APS-related manifestations, laboratory assessment, and details of prior treatment. Patients were divided into two groups, a standard-dose RTX (SD-RTX) group which received a total of more than or equal to 1000mg in 4 weeks and a LD-RTX which received a total of less than 1000mg. LAC was measured by dilute Russell viper venom test (dRVVT) as previously described followed by mixing studies and confirmatory testing when prolonged. Generally, the first step is a sensitive coagulation (dRVVT), the next step is a mixing study and the final confirmatory test involves adding phospholipid, leading to, for example, the dRVVT confirm ratio (17). The titers of aCL (IgA/IgG/IgM) were measured by enzyme linked immunosorbent assay (ELISA) (ORGENTEC, Germany, Product Number: ORG 515S). ACL IgG, IgM and IgA were measured by ELISA (EUROIMMUN, Germany, Product Number: EA 1621-9601 G for IgG, EA 1621-9601 M for IgM and EA 1621-9601 A for IgA). The titers of aβ2GPI (IgA/IgG/IgM) were also measured by ELISA (EUROIMMUN, Germany, Product Number: EA 1632-9601 P). Aβ2GPI IgG, IgM and IgA were measured by ELISA (EUROIMMUN, Germany, Product Number: EA 1632-9601 G for IgG, EA 1632-9601 M for IgM and EA 1632-9601 A for IgA). Venous thromboembolic events (e.g., deep venous thrombosis of the upper limbs of the legs, visceral venous thrombosis, and/or pulmonary embolism) were confirmed by limb ultrasound, pulmonary computed tomography (CT) or scintigraphy (ventilation/perfusion), abdominal pelvic CT scan and vessel angiography as indicated. Arterial thrombotic events (e.g., peripheral arterial thrombosis, acute cerebral infarction, and/or visceral arterial thrombosis) were diagnosed using typical clinical pictures with positive arteriography [e.g., leg or upper limb ultrasound, CT, or magnetic resonance angiography (MRA)] and surgery. The adjusted global antiphospholipid syndrome score (aGAPSS) was calculated for each patient by adding the points corresponding to the risk factors, excluding antibodies to phosphatidylserine/prothrombin (aPS/PT) that are not routinely tested in most clinical laboratories, as previously described (18). The aGAPSS ranged from 0 to 17.

### Follow-up procedure and clinical outcomes

All patients were prospectively followed up after initial RTX administration at month 3 and 6 by the same medical team (YZG and CL). Patients with APS typically require lifelong warfarin anticoagulation following a thrombotic event due to a significant risk of recurrent thrombosis. International normalized ratio (INR) is the preferred test of choice for patients taking warfarin anticoagulant therapy. The INR target is between 2.0 and 3.0 in patients with APS, according to the anticoagulation guidelines of the American College of Chest Physicians (19). Response was evaluated 3 and 6 months after the first dose of RTX. Follow-up information was also obtained from electronic medical records and regular medical examination reports. In accordance with the revised Sapporo criteria (15), complete response (CR) was defined as achieving full resolution of the “indicated manifestation”; partial response (PR) was defined as a favorable response occurred but did not meet the criteria for complete response. Overall response included complete response and partial response. Patients who did not reach remission were considered non-responders (NR). For thrombocytopenia, complete response was defined as a platelet count of > 100×10^9^/L, partial response as 80–100×10^9^/L, and no response as < 80×10^9^/L. For cardiac manifestations, complete response was defined as the disappearance of echocardiographic lesions, partial response as 50% improvement of echocardiographic lesions, and no response as no change or worsening of echocardiographic lesions. For skin ulcer, complete response was defined as disappearance by physical examination and digital imaging, partial response as > 50% improvement, and no response as no change or worsening of skin ulcers. For cognitive dysfunction, complete remission was defined as normalization of the cognitive impairment index with 50% improvement, partial response as abnormal cognitive impairment index with 50% improvement, and no response as no change or worsening of the cognitive impairment index. Adverse events associated with RTX were assessed during drug infusion and throughout follow-up. All adverse events were graded according to the Common Terminology Criteriafor Adverse Events, version 5 (CTCAE) (20).

### Statistical analysis

All statistical analyses were performed using Statistical Product and Service Solutions (SPSS) 25.0 for Windows (IBM, New York, USA). GraphPad Prism version 8.0 were used to produce the graphs. The data were expressed as percentages for categorical variables, mean ± standard deviation (SD) for normally distributed continuous variables, and median [interquartile range (IQR)] for skewedly distributed continuous variables. Differences between LD group and SD group were analyzed by chi-square test or Fisher’s exact as appropriate for categorical variables, and two-tailed independent-sample t test or Mann-Whitney U-test for continuous variables. Differences between LD group and SD group for laboratory manifestations were performed using Kruskal–Wallis tests at baseline and after 3 and 6 months. The cumulative probability of complete response of patients with different treatment dosage groups were drawn using the Kaplan–Meier method. Two-sided *p* < 0.05 was considered statistically significant.

## Results

### Study population and clinical characteristics at baseline

**Figure 1:**
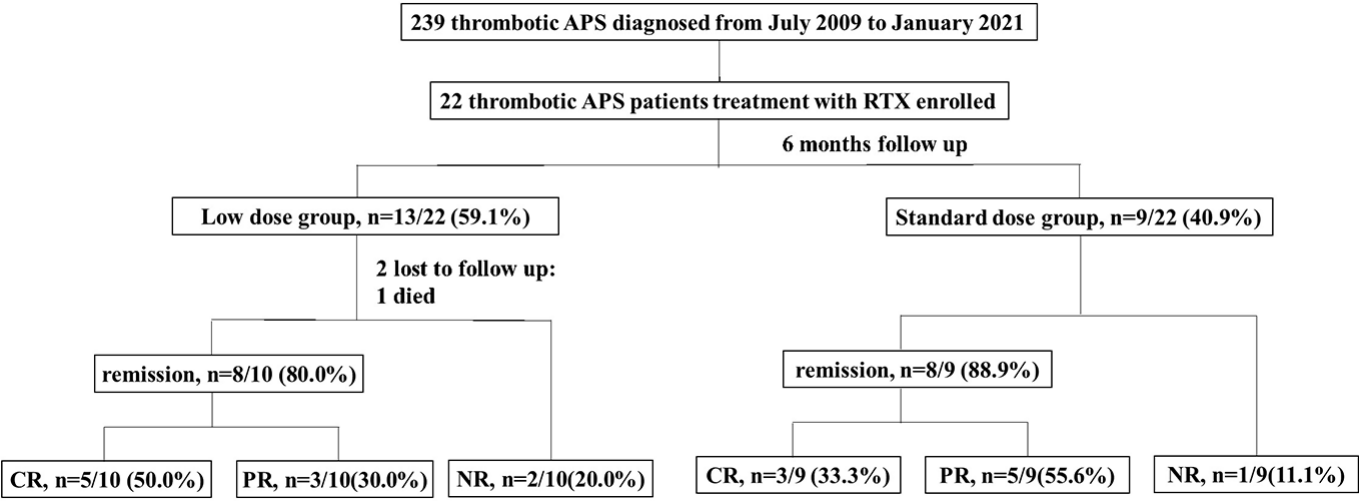
Flow chart of the patients with isolated thrombotic APS receiving rituximab therapy. There were 22 patients enrolled, with 13 patients received low dose rituximab (an overall dosage <1000mg) and 9 patients received standard dose rituximab (an overall dosage ≥1000mg). One patient discontinued treatment because of died, 2 patients lost to follow up, and 19 patients complement within 6-month follow up. The remission rate was 80.0% (50.0% CR, 30.0%PR) in the low dose group and 88.9% (33.3% % CR, 55.6%PR) in the standard dose group. CR, complete remission; PR, partial remission; NR, no remission.

A total of 239 patients with thrombotic APS were enrolled in our cohort, and 22 patients with APS who received RTX as induction therapy. A flow diagram of the individuals at each stage was shown in **Figure 1**. There were a total of 143 courses of RTX. All patients did not receive additional immunosuppressants. The detailed clinical profiles were shown in **Table 1**.

**Table 1:** Detailed clinical profiles of APS patients with rituximab.

No.	Age of onset/gender	aPL profile	Types	Anticoagulation therapy	Non-criteria manifestations	Initial induction therapy	RTX dose (mg)	Maintenance regimen	Outcomes at 6 months
1	45/F	aβ2-GPI, LAC	SAPS	No^&^	Thrombocytopenia	No	2000	Pre	NR
2	72/F	Triple positive	SAPS	Warfarin	Thrombocytopenia	Yes	800	Pre+HCQ	CR
3	24/F	Triple positive	SAPS	Warfarin	Thrombocytopenia	Yes	800	Pre+HCQ	CR
4	30/F	Triple positive	SAPS	No^&^	Thrombocytopenia	No	800	Pre+TAC	NR
5	31/F	LAC	SAPS	Warfarin, INR 1.5-2.0	Thrombocytopenia	No	800	CSA	PR
6	36/F	LAC	SAPS	Warfarin, INR 2.0-3.0	Thrombocytopenia	No	1000	MMF	CR
7	49/M	LAC	CAPS	No^&^	Thrombocytopenia	Yes	600	CSA	NR
8	55/M	aβ2GPI	SAPS	NA^*^	Thrombocytopenia	No	100	NA	NA^*^
9	70/F	aβ2GPI	SAPS	NA^*^	Thrombocytopenia	No	300	NA	NA^*^
10	24/M	aβ2-GPI, LAC	SAPS	Warfarin, INR 2.0-3.0	Thrombocytopenia	Yes	2000	MMF	CR
11	13/F	Triple positive	SAPS	Warfarin	Valvular vegetation	Yes	1000	NA	PR
12	41/F	Triple positive	PAPS	Warfarin, INR 2.0-3.0	Thrombocytopenia	Yes	800	RTX	CR
13	19/M	Triple positive	SAPS	Warfarin, INR 2.0-3.0	Thrombocytopenia, skin ulcer	Yes	1700	RTX	CR
14	53/F	aCL, LAC	SAPS	Warfarin, INR 1.5-2.0	Thrombocytopenia	No	1000	sirolimus	PR
15	49/M	Triple positive	CAPS	Warfarin, INR 2.0-3.0	Thrombocytopenia	No	500	MMF	CR
16	18/F	Triple positive	PAPS	Warfarin, INR 2.0-3.0	/	No	1000	AZA	PR
17	58/F	Triple positive	SAPS	Warfarin, INR 2.0-3.0	Thrombocytopenia	No	800	CSA	CR
18	43/M	Triple positive	SAPS	LMWH	Cognitive dysfunction	No	800	MMF	PR
19	25/M	aCL	PAPS	Warfarin, INR 2.0-3.0	Skin ulcer	No	600	None	PR
20	35/M	Triple positive	SAPS	Warfarin, INR 2.0-3.0	Thrombocytopenia	Yes	2000	RTX	PR
21	39/F	Triple positive	SAPS	Warfarin, INR 2.0-3.0	/	Yes	1000	MMF	PR
22	62/F	Triple positive	SAPS	NA^*^	Thrombocytopenia	No	100	CSA	Died

APS, antiphospholipid syndrome; aβ2GPI, anti-β2-glycoprotein I antibody; aCL, anticardiolipin antibody; LAC, lupus anticoagulant; PAPS, primary antiphospholipid syndrome; SAPS, secondary antiphospholipid syndrome; CAPS, catastrophic antiphospholipid syndrome; Pre, prednisone; RTX, Rituximab; HCQ, hydroxychloroquine; TAC, tacrolimus; CSA, Cyclosporin; MMF, mycophenolate mofetil; AZA, azathioprine; NA, Not available due to lost to follow-up. CR, complete remission, PR, partial remission; NR, no remission; LMWH, low molecular weight heparin.

*Patients lost to follow-up.

^&^Patients did not receive anticoagulation therapy due to sustained severe thrombocytopenia (<20×10^9^/L).

Of 22 patients with APS who received RTX, 13 patients treated with LD-RTX and 9 patients treated with SD-RTX. All patients received hydroxychloroquine during RTX induction therapy. Of 19 patients completed 6-month follow-up, 3 patients did not receive anticoagulation therapy due to sustained severe thrombocytopenia, one patient received LMWH, and 15 patients received warfarin. Of those 15 patients received warfarin, 2 patients did not achieved target INR because of thrombocytopenia (30-50×10^9^/L). The clinical and laboratory characteristics of the patients at baseline were presented in **Table 2**. Patients included in LD-RTX had the characteristics of older age [(49.1 ± 15.5) vs. (35.8 ± 12.3) years, *p* = 0.044] and later-onset [(46.8 ± 16.3) vs. (31.3 ± 13.6) years, *p* = 0.029] than SD-RTX. We next adopted logistics models to test whether there was a relationship between age or age of onset and the dose of RTX, and found there was no significant relationship between age and dose of RTX [OR 1.091 (0.592 - 2.011), *p*=0.087] or age of onset [OR=0.794 (0.465 - 1.354), *p*=0.397]. Gender, clinical or laboratory features, aGAPSS scores and the percentage of RTX usage as initial induction treatment did not show any significant differences between the two groups.

**Table 2:** Baseline characteristics of APS patients with LD-RTX versus SD-RTX.

Variables	Total (n = 22)	Low dose (n = 13)	Standard dose (n = 9)	*p*
Gender (M/F)	8/14	5/8	3/6	1
Age, years	43.6 ± 15.5	49.1 ± 15.5	35.8 ± 12.3	**0.044**
Age of onset, years	40.5 ± 16.8	46.8 ± 16.3	31.3 ± 13.6	**0.029**
Clinical criteria manifestation				
Venous thrombosis, n (%)	14 (63.6)	7 (53.8)	7 (77.8)	0.38
Artery thrombosis, n (%),	12 (54.5)	7 (53.8)	5 (55.6)	1
Arteriovenous thrombosis, n (%)	4 (18.2)	1 (7.7)	3 (33.3)	0.264
Laboratory criteria manifestation				
aβ2 GPI (IgA/IgG/IgM) (RU/mL)	109.9 ± 96.2	117.7 ± 91.5	100.3 ± 106.3	0.699
aβ2-GPI IgA (n=13) (RU/mL)	7.13 (2.07, 43.82)	2.42 (2.04, 104.38)^*^	10.93 (3.06, 50.34)^&^	0.418
aβ2-GPI IgG (n=13) (RU/mL)	16.92 (3.92, 92.70)	15.28 (3.85, 71.56)^*^	23.89 (3.63, 120.07)^&^	0.608
aβ2-GPI IgM (n=13) (RU/mL)	17.24 (2.43, 52.46)	41.33 (9.62, 71.03)^*^	8.21 (2.37, 35.97)^&^	0.341
aβ2 GPI +, n (%)	16 (76.2)	10 (83.3)	6 (66.7)	0.611
aCL (IgA/IgG/IgM) (U/L)	30.8 (13.8, 90.0)	34.9 (14.1, 84.7)	17.3 (13.8, 90.0)	0.943
aCL IgA (n=13) (U/L)	2 (2, 10.64)	2 (2, 61)*	4.76 (2, 10.79)^&^	0.504
aCL IgG (n=13) (U/L)	56.05 (4.98, 100.9)	56.06 (13.76, 97.70)^*^	61.25 (3.51, 110.45)^&^	0.941
aCL IgM (n=13) (U/L)	2.32 (2, 7.81)	2.2 (2, 8.65)^*^	3.65 (2.05, 6.63)^&^	0.824
aCL + (n, %)	15 (71.4)	8 (66.7)	7 (77.8)	0.659
dRVVT screen (s)	56.9 (41.6, 101.7)	54.6 (42.9, 72.3)	83.6 (40.0, 128.6)	0.152
LAC	1.6 (1.4, 2.1)	1.6 (1.4, 1.9)	1.9 (1.4, 2.6)	0.342
LAC+, n (%)	18 (90.0)	10 (90.9)	8 (88.9)	1
Triple positive aPLs, n (%)	13 (59.1)	8 (61.5)	5 (55.6)	1
Laboratory manifestations				
White blood cell count (×10^9^/L)	6.0 (4.2, 9.1)	5.7 (4.8, 9.1)	6.3 (4.0, 6.5)	0.738
Lymphocyte (×10^9^/L)	1.1 ± 0.6	1.1 ± 0.7	1.1 ± 0.5	0.951
Neutrophils (×10^9^/L)	4.1 (2.8, 6.2)	4.1 (2.9, 6.5)	3.8 (2.8, 5.4)	0.664
Hemoglobin (g/L)	117.0 (106.2, 128.0)	116.0 (77.0, 128.0)	117.0 (117.0, 128.0)	0.216
Platelet (×10^9^/L)	66.0 (50.2, 152.0)	55.0 (50.0, 129.2)	104.0 (59.0, 156.0)	0.367
ESR (mm/h)	29.0 (8.0, 63.0)	28.0 (9.0, 75.0)	34.0 (5.0, 39.0)	0.79
CRP (mg/L)	2.5 (1.0, 4.4)	3.2 (2.5, 7.4)	1.0 (0.9, 2.8)	0.075
IgA (g/L)	1.8 (1.2, 3.1)	1.4 (1.1, 2.6)	2.5 (1.2, 4.7)	0.182
IgG (g/L)	12.0 (8.4, 16.8)	12.1 (8.3, 22.9)	11.9 (8.7, 14.6)	0.841
IgM (g/L)	1.1 (0.6, 1.5)	1.2 (0.7, 1.8)	0.6 (0.5, 1.0)	0.102
C3 (g/L)	0.7 (0.5, 0.8)	0.7 (0.5, 0.9)	0.7 (0.6, 0.8)	0.64
C4 (g/L)	0.1 ± 0.1	0.1 ± 0.1	0.2 ± 0.1	0.422
ANA≥1:80, n (%)	15 (68.1)	10 (76.9)	5 (55.6)	0.276
CD19^+^B	56.0 (18.0, 232.0)	20.0 (10.0, 289.0)	63.0 (45.5, 187.5)	0.683
aGAPSS	11.52± 3.70	12.17 ± 3.83	10.67± 3.54	0.371
Initial usage, n (%)	14 (63.6)	8 (61.5)	6 (66.7)	0.584

*Eight patients did not have the data of serum levels of subtypes of aCL and aβ2-GPI.

^&^One patients did not have the data of serum levels of subtypes of aCL and aβ2-GPI.

LD, low-dose; SD, standard dose; RTX, rituximab. APS, antiphospholipid syndrome; M, male; F, female; y, years; aβ2GPI, anti-β2-glycoprotein I antibody; aCL, anticardiolipin antibody; dRVVT, dilute Russell viper venom test; LAC, lupus anticoagulant; aPL, antiphospholipid antibody; CRP, C reactive protein; ESR, erythrocyte sedimentation rate; IgA, Immunoglobulin A; IgG, Immunoglobulin G; IgM, Immunoglobulin M; C3, Complement 3; C4, Complement 4; aGAPSS, adjusted Global Antiphospholipid Syndrome Score. The bold values mean statistical significance.

### Comparison of treatment response between standard - and low-dose RTX groups at 6 months

Follow-up data were available for 20 patients (90.1%), and one of them died within 1 month. Of 19 patients who completed 6-month follow-up, 8 patients (42.1%) achieved complete response. Following rituximab treatment, the levels of aPLs, immunoglobulin, C reactive protein (CRP) and erythrocyte sedimentation rate (ESR) were assessed at 3 months and 6 months post therapy (**Figure S1**). None of the above parameters showed significant changes after 3 months. After 6 months, a significant decrease of aCL titers {including aCL IgA/IgG/IgM (U/L) [30.8 (10.7,90) vs. 19.50 (2.45,69.10), *p*=0.023] and aCL IgG (U/L) [75.39 (4.98,120) vs. 54.21(2,73.04), *p*=0.043]} and ESR (mm/h) [29 (6, 63) vs. 6 (3, 14), *p*=0.021] was observed, and other parameters, including aβ2-GPI, LAC, immunoglobulin and CRP did not show any significant decrease (**Table 3** **)**.

**Table 3:** Profiles of laboratory parameters following rituximab treatment.

Parameters	Pre-treatment	Post-treatment (n=19)			
		3 months		6 months	
		Value	p*	Value	p*
aβ2 GPI (IgA/IgG/IgM) (RU/mL)	76.27 (17.07,189.10)	137.13 (37.03, 267.10)	0.500	13.66 (3.46,132.49)	0.084
aβ2-GPI IgA (RU/mL)	6.01 (2.04,32)	101.04 (1.55,151.26)^&^	0.180	11.74 (2.33,73.07)^&^	0.893
aβ2-GPI IgG (RU/mL)	15.28 (5.12,141.50)	57.67 (4.28,83.49)^&^	0.655	23.89 (2,44.04)^&^	0.225
aβ2-GPI IgM (RU/mL)	40.92 (12.55,100.36)	29.29 (12.93,32.25)^&^	0.180	0.43 (2,25.23)^&^	0.225
aCL (IgA/IgG/IgM) (U/L)	30.8 (10.7,90)	30.10 (2.60,75.90)	0.735	19.50 (2.45,69.10)	**0.023**
aCL IgA (U/L)	2 (2,6.47)	61 (1.5,91.26)	0.317	4.76 (2,21.55)	0.655
aCL IgG (U/L)	75.39 (4.98,120)	72.76 (19.13,91.26)	0.665	54.21(2,73.04)	**0.043**
aCL IgM (U/L)	5.02 (2.16,9.76)	5.31 (1.5,7.71)	0.180	2.2 (2,5.88)	0.686
dRVVT screen (s)	56.9 (41.6, 101.7)	43.3 (40, 80.5)	0.612	56.3 (51.3, 75.1)	0.333
LAC	1.61 (1.37,2.17)	1.41 (1.12,2.15)	0.833	1.62 (1.49,2.06)	0.202
ESR (mm/h)	29 (6, 63)	14 (8, 23)	0.176	6 (3, 14)	**0.021**
CRP (mg/L)	2.49 (0.95,5.80)	1.68 (0.50,2.41)	0.674	1.51 (0.50,5.03)	0.678
IgA (g/L)	1.80 (1.15,3.37)	2.29 (1.42,3.86)	0.260	2.06 (1.37,3.85)	0.363
IgG (g/L)	12 (8.38,18.25)	11.20 (8.48,15.45)	0.173	10.45 (8.69,11.93)	0.140
IgM (g/L)	1.07 (0.52,1.54)	0.81 (0.37,1.54)	0.441	0.71 (0.31,1.24)	0.124
C3 (g/L)	0.67 (0.46,0.86)	0.78 (0.70,0.93)	0.173	0.98 (0.69,1.10)	0.177
C4 (g/L)	0.13 (0.10,0.16)	0.16 (0.12,0.20)	0.109	0.20 (0.15,0.24)	0.100
aGAPSS	11 (7, 13)	11 (7, 13)	0.344	8 (3.25,13.75)	0.107

^&^Six patients did not have the data of serum levels of subtypes of aCL and aβ2-GPI.

aβ2GPI, anti-β2-glycoprotein I antibody; aCL, anticardiolipin antibody; dRVVT, dilute Russell viper venom test; LAC, lupus anticoagulant; ESR, Erythrocyte sedimentation rate; CRP, C reactive protein; IgA, Immunoglobulin A; IgG, Immunoglobulin G; IgM, Immunoglobulin M; C3, Complement 3; C4, Complement 4; aGAPSS, adjusted Global Antiphospholipid Syndrome Score.

*Comparison with parameters at baseline. The bold values mean statistical significance.

**Figure 2:**
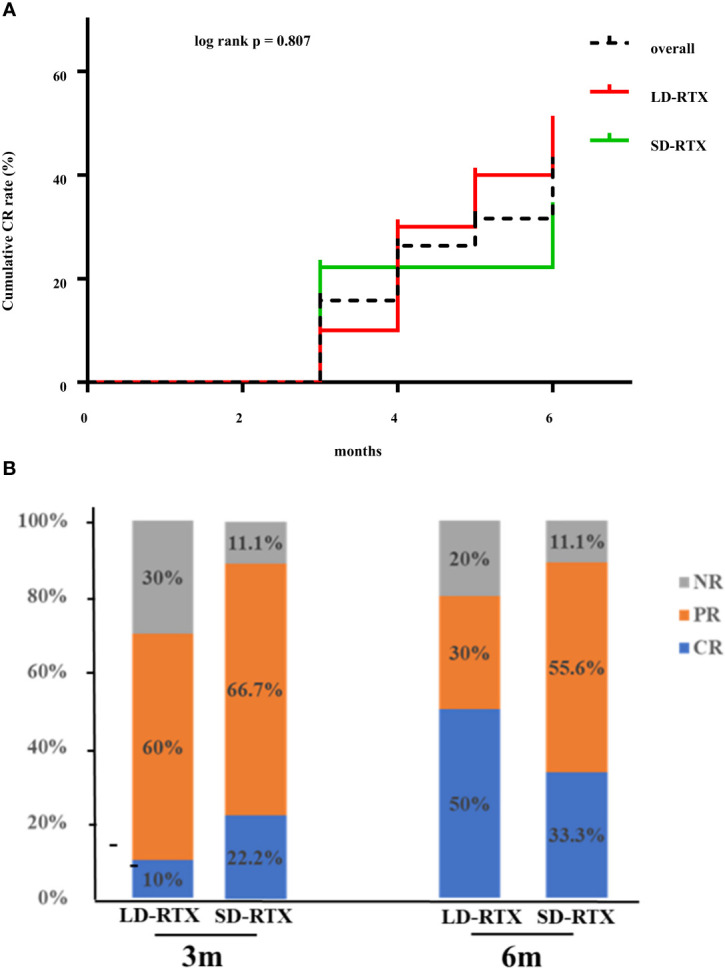
Remission rate at 6 months. **(A)** Cumulative complete remission rate for 6 months after induction therapy between patients with SD-RTX and LD-RTX. **(B)** Comparison of remission rate between patients with SD-RTX and LD-RTX at 3 months and 6 months. SD-RTX, standard-dose rituximab; LD-RTX, low-dose rituximab; CR, complete remission; PR, partial remission; NR, no remission.

Cumulative CR rates were compared between the two groups (**Figure 2A**). There was no significant difference in CR rate for 6 months between the groups (SD-RTX 33.3% vs. LD-RTX 50%, log-rank, *p* = 0.807). We also compared the numbers of patients with CR, PR and NR **(** **Figure 2B** and **Table S1** **)** and laboratory parameters **(** **Table 4** **)** between the two groups. Apart from the levels of IgA were significantly lower in LD-RTX group than SD-RTX group [(1.95 ± 0.26) vs. (2.80 ± 0.21) g/L, p=0.03], there were no significant differences in different status of remission rate and laboratory parameters between SD-RTX group and LD-RTX group at 3 months and 6 months.

**Table 4:** Comparison of laboratory manifestations in APS patients between low dose versus standard dose at 3 months and 6 months follow up.

Parameters	3 months follow up			6 months follow up		
	Low dose (n=10)	Standard dose (n=9)	*p*	Low dose (n=10)	Standard dose (n=9)	*p*
aβ2 GPI (IgA/IgG/IgM) (RU/mL)	121.61 ± 9.03	134.81 ± 4.63	0.417	87.56 ± 16.17	63.51 ± 15.67	0.317
aCL (IgA/IgG/IgM) (U/L)	25.66 ± 5.25	48.18 ± 6.12	0.068	0.75 ± 0.23	0.58 ± 0.22	0.613
dRVVT screen (s)	50.06 ± 4.57	59.08 ± 5.28	0.287	55.67 ± 24.62	81.13 ± 17.82	0.434
LAC	1.60 ± 0.16	1.53 ± 0.21	0.805	1.59 ± 0.29	1.92 ± 0.23	0.413
ESR (mm/h)	19.20 ± 7.92	14.18 ± 6.86	0.664	9.43 ± 4.81	10.05 ± 4.34	0.928
CRP (mg/L)	1.63 ± 0.61	4.08 ± 2.42	0.370	1.30 ± 1.42	1.86 ± 1.05	0.760
IgA (g/L)	2.76 ± 0.29	2.87 ± 0.20	0.772	1.95 ± 0.26	2.80 ± 0.21	**0.030**
IgG (g/L)	14.25 ± 2.20	11.89 ± 1.62	0.427	8.95 ± 0.98	11.46 ± 0.85	0.081
IgM (g/L)	1.19 ± 0.12	0.89 ± 0.11	0.135	1.11 ± 0.29	0.97 ± 0.41	0.785
C3 (g/L)	0.69 ± 0.05	0.80 ± 0.05	0.192	0.76 ± 0.09	1.01 ± 0.08	0.066
C4 (g/L)	0.12 ± 0.02	0.19 ± 0.02	0.082	0.16 ± 0.03	0.24 ± 0.03	0.079

aβ2GPI, anti-β2-glycoprotein I antibody; aCL, anticardiolipin antibody; LAC, lupus anticoagulant; ESR, Erythrocyte sedimentation rate; CRP, C reactive protein; IgA, Immunoglobulin A; IgG, Immunoglobulin G; IgM, Immunoglobulin M; C3, Complement 3; C4, Complement 4; aGAPSS, adjusted Global Antiphospholipid Syndrome Score. The bold values mean statistical significance.

### Safety and adverse reactions

Adverse events during 6 month after RTX initiation were summarized in **Table 5**. In our cohort, serious adverse events were not reported. There were no significant differences between the two groups in the number of adverse events. In LD-RTX group, 1 patient developed pulmonary infection and 1 patient present with elevated liver enzyme post therapy that resolved spontaneously. In SD-RTX group, infusion reactions were documented in 1 patient who were treated with anti-histamines and glucocorticoids with no need to change the protocol. One patient died of macrophage activation syndrome.

**Table 5:** Adverse events at 6 months.

Variable	SD-RTX (n=9)	LD-RTX (n=11)*	*p*
Death, n (%)	0	1 (9.1)	0.45
Total number of selected events, n (%)	1 (11.1)	2 (18.2)	1
Cancer, n (%)	0	0	/
Infection, n (%)	0	1 (9.1)	0.45
Liver function impairment, n (%)	0	1 (9.1)	0.45
Infusion reaction, n (%)	1 (11.1)	0	0.45

SD-RTX, standard-dose rituximab; LD-RTX, low-dose rituximab.

*Two patients lost to follow-up.

## Discussion

In this study, we found the levels of aCL and ESR decreased significantly after RTX treatment, and there were no significant differences in CR rate, aβ2GPI titers, LAC or adverse events for 6 months between patients taking low and standard doses of RTX for APS. Our findings suggested that low-dose treatment with RTX might be an alternative choice for elderly patients with APS as an induction therapy. To our knowledge, this is the first study to discuss the efficacy and safety of low-dose RTX in Asian patients with APS.

B cells, notably through aPLs production, play a key role in the development of APS (21). RTX is a chimeric monoclonal antibody targeting CD20 and can reduce cytokine secretion and autoantibody production by specifically targeting B cells expressing CD20 (22). Nowadays, RTX has been confirmed as an effective B cell depletion therapy in various autoimmune disorders, including APS (21, 22). Previous pilot studies have discovered favorable results of RTX in APS that a majority of APS patients achieved clinical improvement, especially non-criteria manifestations (8, 9, 23). A multicenter retrospective study revealed 55% refractory APS achieved CR following either 2 doses of 1000mg RTX (2 weeks apart) or 4 doses of 375mg/m^2^ (once weekly) (7). Similarly, we found nearly half patients achieved complete remission after RTX during 6-month follow-up. Given all this evidence, application of RTX in APS contributes to decrease of aPLs and disease remission.

aPLs are mainly produced by plasma cells and circulating CD20 negative B cells (e.g. plasmablasts) (7, 24). Since RTX has no effect on memory B cells or long-lived plasma cells, the titers of aPLs might not show substantial change after RTX treatment, which has been proved by a phase II trial (8). However, our study revealed a significant decrease of aCL titers after RTX therapy, consistent with Ioannou Y et al’s findings (25). Besides, Yang et al. found that after RTX, the titers of aCL decreased within 1-year follow-up and a substantial decrease of the titers of aβ2-GPI was observed within 2-year follow-up even after the recovery of B cells (6).These observations imply that different kinds of aPLs might have different mechanisms of production, and aCL might be mainly secreted by short-lived plasma cells in certain populations (24, 25). Moreover, some previous studies also showed significant decrease of aPL titers after RTX therapy (6, 7, 23). Apart from producing antibody directly, B cells have other immune functions, such as promoting antigen recognition and activation of T cells, or modulate immune response by secreting cytokines, which could also regulate autoantibody production indirectly. To sum up, it seems that at least for some patients, a decrease in aPL titres, within a certain period of time following rituximab treatment, is associated with a favorable outcome and may possibly be used as a marker for response to therapy. Other immune modulating mechanisms, independent of autoantibody production may also be associated with a clinical response to rituximab treatment. Therefore, different response of RTX treatment represents different populations, different methods of aPL detection or even different timing of aPL sampling.

Even if RTX has a relatively good safety profiles, immunosuppression, infusion reactions, and hepatitis virus/mycobacterial reactivations can occur (26). Besides, the B-cell total load in patients with APS is much less than that in patients with lymphoma. Furthermore, since RTX is expensive ($4912.79 in the USA or ¥7866.26 in PRC, per 500 mg) (14). Therefore, a reduced dosage of rituximab might still be sufficient for its therapeutic purpose with socioeconomically preference in preventing disease flare or suppressing disease activity.

In our study, we also found LD-RTX was an efficacious and safe treatment as SD-RTX for APS in decreasing titers of aPLs and reducing disease activity. LD-RTX has been shown similar efficacy to those successful results obtained with standard-dose regimens in autoimmune diseases (14, 27–29). For example, in rheumatoid arthritis (RA), Chatzidionysiou K et al. reported no significant difference was seen in the percentages of patients who achieved a European League Against Rheumatism good response at 6 months between high- (two doses of 1000 mg) and low-dose RTX groups (two doses of 500 mg) (29). In addition, in our study cohort, we found elder patients with APS tended to receive LD-RTX as induction therapy. Such tendency is also found in RA and ANCA-associated vasculitis (AAV) (14, 29). Furthermore the pharmacokinetics of RTX was highly variable among patients with AAV despite a dosing protocol that adjusted for the body surface area, and higher RTX exposure was not associated with important clinical outcomes (30). All these findings support our findings that SD-RTX might not be necessary for all the APS patients and some could be treated with LD-RTX, especially elder patients.

### Study limitation

This study has several limitations. Firstly, due to the retrospective design of a single-center study, only patients who could be observed for more than 6 months were enrolled, which induced a degree of selection bias. Besides, T cell and B cell counts are not routinely tested in our center, thus we could not discuss the different effect on B cell depletion between LD-RTX and SD-RTX. Future prospective or multicenter studies are desired to validate our findings.

### Conclusion

In the present study, we found that RTX might be effective in reducing aPL production and controlling disease activity, and LD-RTX may be as efficacious as SD-RTX in induction therapy for APS.

## Data availability statement

The original contributions presented in the study are included in the article/**Supplementary Material**. Further inquiries can be directed to the corresponding author.

## Ethics statement

The studies involving human participants were reviewed and approved by the ethics committee of Peking University People’s Hospital. The patients/participants provided their written informed consent to participate in this study. Written informed consent was obtained from the individual(s) for the publication of any potentially identifiable images or data included in this article.

## Author contributions

YG and XZ: data interpretation and analysis, writing of the original draft, review, and editing. GL and YZ: clinical data collection. HY: editing and follow-up of participants. CL: conceptualization, methodology, investigation, resources, data curation, supervision, manuscript editing, and funding acquisition. The authors have read and approved the final manuscript.

## Funding

This work was supported by the National Natural Science Foundation of China (No. 81801615, 81871289, No. 82071814), University of Michigan Medical School (UMMS) and Peking University Health Science Center (PUHSC) Joint Institute (JI) Projects (No. BMU2020JI003), Peking University Medicine Fund of Fostering Young Scholars’ Scientific & Technological Innovation and Fundamental Research Funds for the Central Universities (BMU2022PY004), and Peking University People’s Hospital Research and Development Funds (RDY 2019-04).

## Acknowledgments

The authors thank Dr. Huixin Liu for her support with the statistics of the project.

## Conflict of interest

The authors declare that the research was conducted in the absence of any commercial or financial relationships that could be construed as a potential conflict of interest.

## Publisher’s note

All claims expressed in this article are solely those of the authors and do not necessarily represent those of their affiliated organizations, or those of the publisher, the editors and the reviewers. Any product that may be evaluated in this article, or claim that may be made by its manufacturer, is not guaranteed or endorsed by the publisher.
