# Supplementary material for: Low dose versus standard dose rituximab for the treatment of antiphospholipid syndrome: A pilot study from a tertiary medical center
Source: Front Immunol. 2022 Nov 3;13:971366. doi: 10.3389/fimmu.2022.971366 (PMC9670802; doi:10.3389/fimmu.2022.971366)
Supplement: Supplementary file 1 [file DataSheet_1.docx]

**Table S1. APS response to rituximab in patients with low dose versus standard dose**

|  | Parameters | Total  (n=19) | Low dose  (n=10) | Standard dose  (n=9) | *p* |
| --- | --- | --- | --- | --- | --- |
| 3m | Complete response, n(%) | 3 (15.8) | 1 (10) | 2 (22.2) | 0.666 |
|  | Partial response, n(%) | 12 (63.2) | 6 (60) | 6 (66.7) |  |
|  | No responder, n(%) | 4 (21.1) | 3 (30) | 1 (11.1) |  |
| 6m | Complete response, n(%) | 8 (42.1) | 5 (50) | 3 (33.3) | 0.586 |
|  | Partial response, n(%) | 8 (42.1) | 3 (30) | 5 (55.6) |  |
|  | No responder, n(%) | 3 (15.8) | 2 (20) | 1 (11.1) |  |


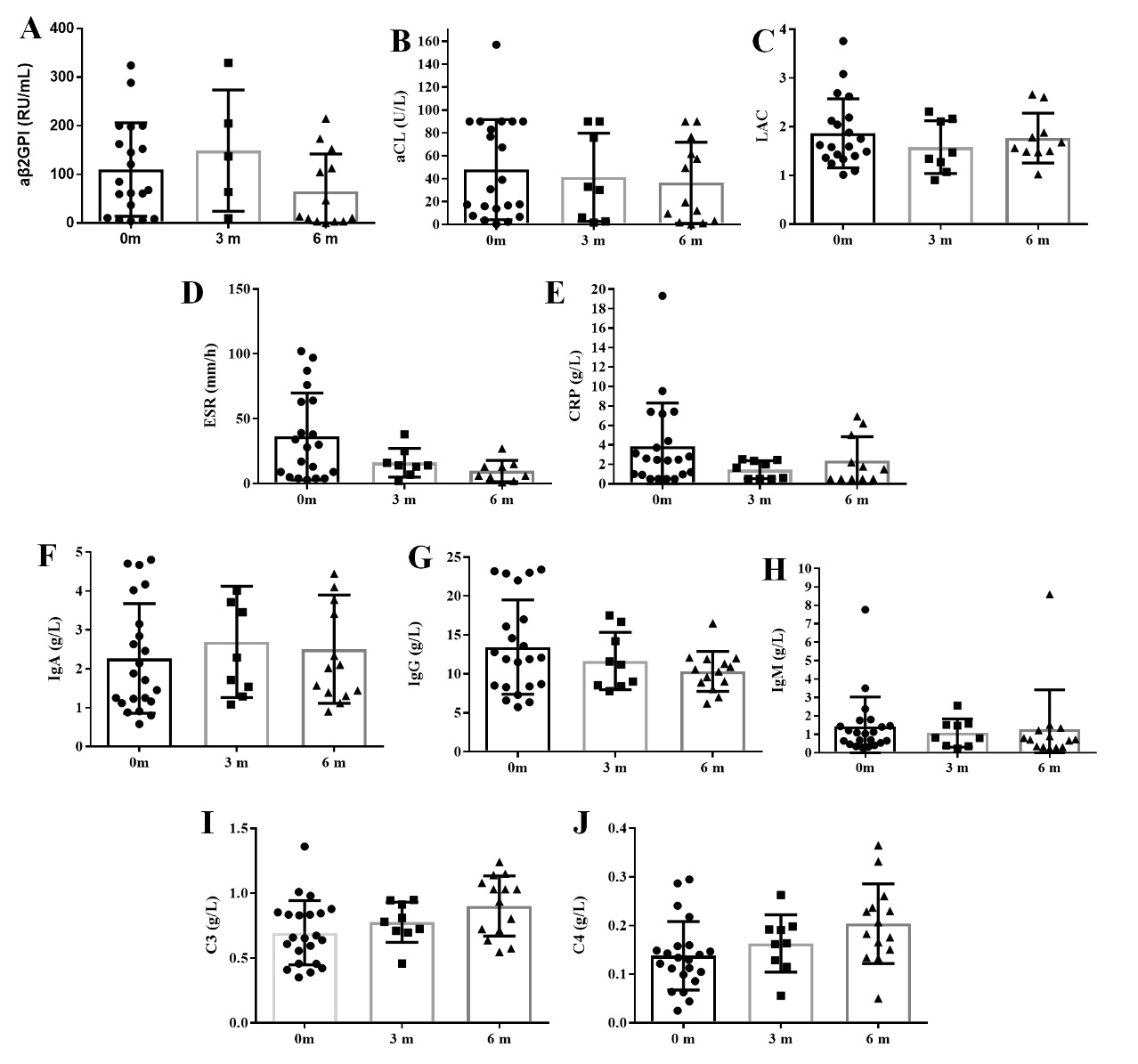


Figure S1. Efficacy of RTX in patients with antiphospholipid syndrome. (A) aβ2GPI, anti-β2-glycoprotein I antibody; (B) aCL, anticardiolipin antibody; (C) LAC, lupus anticoagulant; (D) ESR, erythrocyte sedimentation rate; (E) CRP, C reactive protein; (F) IgA, Immunoglobulin A; (G) IgG, Immunoglobulin G; (H) IgM, Immunoglobulin M; (I) C3, Complement 3; (J) C4, Complement 4.
